# Elevated transforming growth factor β and mitogen-activated protein kinase pathways mediate fibrotic traits of Dupuytren's disease fibroblasts

**DOI:** 10.1186/1755-1536-4-14

**Published:** 2011-06-28

**Authors:** Carola Krause, Peter Kloen, Peter ten Dijke

**Affiliations:** 1 Department of Molecular Cell Biology and Centre for Biomedical Genetics, Leiden University Medical Center, Einthovenweg 20, 2333 ZC Leiden, The Netherlands; 2 Institute for Chemistry and Biochemistry, Freie Universität Berlin, Thielallee 63, D-14195 Berlin, Germany; 3 Department of Orthopedic Surgery, Academic Medical Center, Meibergdreef 9, 1100 DD, Amsterdam, The Netherlands

## Abstract

**Background:**

Dupuytren's disease is a fibroproliferative disorder of the palmar fascia. The treatment used to date has mostly been surgery, but there is a high recurrence rate. Transforming growth factor β (TGF-β) has been implicated as a key stimulator of myofibroblast activity and fascial contraction in Dupuytren's disease.

**Results:**

We studied Dupuytren's fibroblasts in tissues *ex vivo *and in cells cultured *in vitro *and found increased TGF-β expression compared to control fibroblasts. This correlated not only with elevated expression and activation of downstream Smad effectors but also with overactive extracellular signal-regulated kinase 1/2 (ERK1/2)/mitogen-activated protein (MAP) kinase signalling. Treatment with the TGF-β type I receptor kinase inhibitor SB-431542 and bone morphogenetic protein 6 (BMP6) led to inhibition of elevated Smad and ERK1/2/MAP kinase signalling as well as to inhibition of the increased contractility of Dupuytren's fibroblasts. BMP6 attenuated TGF-β expression in Dupuytren's fibroblasts, but not in control fibroblasts. Platelet-derived growth factor (PDGF) expression was strongly promoted by TGF-β in Dupuytren's fibroblasts and was curbed by SB-431542 or BMP6 treatment. High basal expression of phosphorylated ERK1/2 MAP kinase and fibroproliferative markers was attenuated in Dupuytren's fibroblasts by a selective PDGF receptor kinase inhibitor. Cotreatment of Dupuytren's fibroblasts with SB-431542 and the mitogen-activated protein kinase kinase 1 inhibitor PD98059 was sufficient to abrogate proliferation and contraction of Dupuytren's fibroblasts.

**Conclusions:**

Both TGF-β and ERK1/2 MAP kinase pathways cooperated in mediating the enhanced proliferation and high spontaneous contraction of Dupuytren's fibroblasts. Our data indicate that both signalling pathways are prime targets for the development of nonsurgical intervention strategies to treat Dupuytren's disease.

## Background

Dupuytren's disease (DD) is a common fibroproliferative condition that only affects the hand. The characteristic feature is a progressive contracture of the palm and fingers. Patients commonly first display a nodule in the palm or on the volar (palmar) aspect of the fingers caused by a thickened layer of tissue (palmar fascia) between the skin and the tendons of the hand and fingers. The nodule is a key diagnostic feature and represents the early proliferative stage of the disease. The nodules contain mostly myofibroblasts [1,2]. As the disease progresses, the nodules may disappear and give way to the formation of cords. These cords represent characteristics of fibrosis within the involutional and residual stages of the disease and comprise mostly fibroblasts and extracellular matrix (ECM).

Treatment of DD consists largely of surgical excision of the contracted tissue. Because of high recurrence rates following surgery, investigations are underway to determine the underlying causes of DD to optimise treatment strategies [1,2]. The myofibroblast, a specialised fibroblast phenotype that expresses α-smooth muscle actin (α-SMA), provides the cell with contractile activity [3-5]. To date, many growth factors have been implicated in Dupuytren's contracture; transforming growth factor β (TGF-β) in particular has been proposed to play a prominent role [6].

TGF-β is a member of a protein family that also includes activins, nodal and bone morphogenetic proteins (BMPs). TGF-β protein family members signal through type I and type II serine/threonine kinase receptors [7]. Type I receptors are also called 'activin receptor-like kinases' (ALKs). ALK4, ALK5 and ALK7 are type I receptors of activin, TGF-β and nodal protein kinases, respectively. SB-431542 is a selective inhibitor of ALK4, ALK5 and ALK7 kinase activity [8]. Signalling from activated type I receptors is mainly transduced into the cytoplasm through phosphorylation of receptor-regulated Smads (R-Smads). Activated ALK4, ALK5 and ALK7 induce phosphorylation of Smad2 and Smad3. BMPs mediate the activation of Smad1, Smad5 and Smad8. Activated R-Smads form heteromeric complexes with Smad4 that accumulate in the nucleus, where they regulate gene expression, including plasminogen activator inhibitor 1 (PAI-1; also known as SERPINE1, a TGF-β/ALK5 target gene) and the inhibitor of DNA binding 1 gene (a BMP target gene) [7]. TGF-β can also activate non-Smad pathways, including the extracellular signal-regulated kinase (ERK) mitogen-activated protein (MAP) kinase signalling pathway [9,10]. TGF-β is a potent modulator of fibroblast and myofibroblast proliferation and differentiation [3,11-13]. Previous studies of DD tissue found increased protein synthesis and expression of all three TGF-β isoforms and their receptors [14-18]. *In vitro *contraction assays revealed that TGF-β stimulation generates or increases contractile force in Dupuytren-derived cells [19-23]. In addition, TGF-β stimulation leads to upregulation of key ECM components, such as fibronectin and type I collagen (COL1), and this effect either may be direct or may occur indirectly via enhanced expression of matricellular protein connective tissue growth factor (CTGF/CCN2) [24,25]. TGF-β stimulation can also induce the expression of growth factors, such as platelet-derived growth factor (PDGF) [26].

It is not known whether BMPs play a role in DD. Compared to normal fascia-derived cells, Dupuytren-derived cells do not express BMP4 and exhibit decreased BMP6 and BMP8 expression [27]. A previous study found that there is decreased BMP receptor expression and, apparently, reduced BMP responsiveness in DD tissue, which has constrained research into BMPs as potential antagonists of TGF-β-induced fibrosis in DD as described in kidney and liver fibrosis [28].

In this study, we investigated the aberrant activation of the TGF-β/Smad and PDGF/ERK1/2/MAP kinase pathways in DD tissue specimens and cell culture. Using BMP6 and selective chemical inhibitors of the TGF-β receptors, the PDGF receptors and the MAP kinase pathway, we attempted to counteract the fibrogenic characteristics of DD. Our insights may contribute to the development of new therapeutic strategies for sustained, nonsurgical treatment of DD.

## Methods

### Clinical specimens

DD tissue specimens were obtained from four adult patients undergoing fasciectomy for DD. Patients who underwent carpal tunnel release and showed no evidence of DD contributed the control tissue from normal palmar fascia (*n *= 3) or carpal ligament (*n *= 1). All DD tissues used were from primary releases. The tissue was separated macroscopically in nodules and cords. Only nodules were used in this study. For details on how samples were prepared, see the Additional files.

### Clinical sample preparation

After excision, the nodule was divided in three portions. One portion was placed in 10% formalin and further processed for immunohistochemistry. The second was immediately placed into liquid nitrogen for protein extraction. The third portion was used for primary cell culture. All of the patients underwent excision independently of this study and had not undergone previous surgery on their hands. Oral consent for removal of the tissue and storage in the tissue bank for research purposes was obtained from the patients. Individual consent for this specific project was waived by the local ethics committee because the research was performed on 'waste' material, which was stored in a coded fashion.

### Reagents

Recombinant human TGF-β3 (OSI Pharmaceuticals Inc., Melville, NY, USA) and recombinant human BMP6 (Creative BioMolecules, Hopkinton MA, USA) were generously provided by K Iwata and K Sampath, respectively. SB-431542 compound, which targets ALK4, ALK5 and ALK7, was purchased from Tocris Bioscience, Ellisville, Missouri, USA. The PD98059 compound, which targets mitogen-activated protein kinase kinase 1 (MEK1), was purchased from Cell Signaling Technology (Danvers, MA, USA). The vascular endothelial growth factor (VEGF) receptor inhibitor PTK787/ZK222584, the epidermal growth factor (EGF) receptor inhibitor PKI166 and the PDGF receptor inhibitor STI571 (also known as imatinib mesylate) were kindly provided by Novartis, Amsterdam, The Netherlands. The protein kinase C activator 12-*O*-tetradecanoyl-phorbol-13-acetate (TPA) was obtained from Sigma (Sigma Chemical Co., St. Louis, MO, USA).

### Cell culture

To obtain primary cells, tissues were minced under sterile conditions into pieces that measured approximately 1 × 1 × 2 mm^3^. Ten to twenty pieces were placed as explants into the wells of six-well plates and stored in 37°C incubators in 5% CO_2_. Primary cells from passages 3 through 6 were used for the experiments. All of the cells were subcultured in DMEM containing 4.5 g/L glucose (Gibco, Breda, The Netherlands) supplemented with 10% foetal bovine serum (FBS) (Integro, Zaandam, The Netherlands), 100 IU/mL penicillin and 100 IU/mL streptomycin (Invitrogen, Breda, The Netherlands).

### RNA isolation and quantitative real-time PCR

Total RNA was extracted by using the RNeasy Kit (Qiagen, Venlo, The Netherlands) according to the manufacturer's instructions. Reverse transcriptase PCR was performed using the RevertAid H Minus First Strand cDNA Synthesis Kit (Fermentas, St. Leon-Rot, Germany) according to the manufacturer's instructions. All of the samples were plated in duplicate, and TaqMan PCR reactions were performed using the StepOnePlus Real-Time PCR System (Applied Biosystems, Carlsbad, California, USA). Lack of DNA contamination was verified and gene expression levels were determined using the comparative Δ*C*_t _method with glyceraldehyde 3-phosphate dehydrogenase (*GAPDH*) as the reference.

### Quantitative PCR primers

Human *TGF-β1 *through *TGF-β3, α-SMA, PAI-1, c-myc, COl1A2, fibronectin, Smad1 *through *Smad3, CTGF, PDGF-A, PDGF-B *and *GAPDH *gene expression was analysed using the following forward and reverse primers: *TGF-β1*, 5'-CTCTCCGACCTGCCACAGA-3' and 5'-AACCTAGATGGGCGCGATCT-3'; *TGF-β2*, 5'-CCGCCCACTTTCTACAGACCC-3' and 5'-GCGCTGGGTGGGAGATGTTAA-3'; *TGF-β3*, 5'-CTGGCCCTGCTGAACTTTG-3' and 5'-AAGGTGGTGCAAGTGGACAGA-3'; *α-SMA*, 5'-CACCTTCCAGCAGATGTGGAT-3' and 5'-AAGCATTTGCGGTGGACAAT-3'; *PAI-1*, 5'-TCTTTGGTGAAGGGTCTGCT-3' and 5'-CTGGGTTTCTCCTCCTGT TG-3'; *c-myc*, 5'-CGTCTCCACACATCAGCACAA-3' and 5'-CACTGTCCAACTTGACCCTCTTG-3'; *COl1A2*, 5'-GATGTTGAACTTGTTGCTGAGG-3' and 5'-TCTTTCCCCATTCATTTGTCTT-3'; *fibronectin*, 5'-GAGGCCACCATCACTGGTT-3' and 5'-AGTGCGATGACATAGATGGTGTA-3'; *Smad1*, 5'-TGAACCATGGATTTGAGACAGT-3' and 5'-CTGGCGGTGGTATTCTGC-3'; *Smad2*, 5'-CGAAAAGGATTGCCACATGTT-3' and 5'-TTGAGTTCATGATGACTGTGAAGATC-3'; *Smad3*, 5'-CGGTCAACCAGGGCTTTG-3' and 5'-CAGCCTTTGACGAAGCTCATG-3'; *CTGF*, 5'-TTGCGAAGCTGACCTGGAAGAGAA-3' and 5'-AGCTCGGTATGTCTTCATGCTGGT-3'; *PDGF-A*, 5'-CCTCACATCCGTGTCCTCTT-3' and 5'-ACACGAGCAGTGTCAAGTGC-3'; *PDGF-B*, 5'-TGCTGTTGAGGTGGCTGTAG-3' and 5'-GAAAATGCAGGGTGGAGGTA-3'; *TGF-α*, 5'-TAACCACGAGACCCTCAACC-3' and 5'-CCCAAGCCTTAGCTGTCTTG-3'; and *GAPDH*, 5'-ATCACTGCCACCCAGAAGAC-3' and 5'-ATGAGGTCCACCACCCTGTT-3'.

### MTS-based proliferation assay

Cells were seeded into 96-well plates at 7 × 10^3 ^cells/well and treated the next day with the indicated inhibitors or DMSO as a control. Increases in the number of viable cells after culture were measured daily for 4 days using an 3-(4,5-dimethylthiazol-2-yl)-5-(3-carboxymethoxyphenyl)-2-(4-sulfophenyl)-2H-tetrazolium (MTS)-based proliferation assay according to the manufacturer's instructions (CellTiter 96 AQueous One Solution Cell Proliferation Assay System; Promega, Leiden, The Netherlands) and using the measured absorbance at 490 nm on day 0 as the reference.

### Tissue lysate preparation and Western blot analysis

For tissue lysates, biopsies were frozen in liquid nitrogen and pulverised using a mortar. Thereafter, the triturated tissues were incubated in ice-cold lysis buffer (150 mmol NaCl, 20 mmol Tris·HCl, pH 7.5, 1% Nonidet P-40, 5 mmol sodium ethylenediaminetetraacetic acid (EDTA) and one Complete Protease Inhibitor Cocktail Tablet (Roche, Woerden, The Netherlands) per 50 mL of solution) for 30 minutes. Prior to centrifugation at 4°C for 15 minutes at 14 × 10^3 ^rpm, the samples underwent extensive vortexing and sonification. The total protein content of the supernatant was determined using the DC Protein Assay (Bio-Rad Laboratories, Veenendaal, The Netherlands)). Equal amounts of total protein (100 μg/μL) were loaded onto a 10% gel, followed by SDS-PAGE and Western blot analysis. For cell-based assays, cells were plated onto six-well plates at a density of 4 × 10^5 ^cells/well, stimulated with the indicated reagents and directly lysed in sample buffer (250 mmol Tris·HCl, pH 6.8, 8% SDS, 40% glycerol, 5% β-mercaptoethanol, and bromophenol blue) after 18 hours. Antibodies specifically targeting Smad1 (Zymed, San Francisco, CA, USA), Smad2/3 (BD Transduction Laboratories, Breda, The Netherlands), phosphorylated ERK1/2 (P-ERK1/2; Cell Signaling Technology), PAI-1 (Santa Cruz Biotechnology, Santa Cruz, CA, USA), Col1α 2 (Col1A2; SouthernBiotech, Birmingham, Alabama, USA), α-SMA (Sigma Chemical Co.), fibronectin/ED-A (Abcam, Cambridge, MA, USA) and c-myc (Santa Cruz Biotechnology) were purchased. Antibodies targeting phosphorylated Smad1 and Smad2 (P-Smad1 and P-Smad2, respectively) were described previously [29]. P-Smad3 was obtained from E Leof (Mayo Clinic, Rochester, MN, USA), and P-ERK1/2 antibodies were a gift from WH Moolenaar (Netherlands Cancer Institute, Amsterdam, The Netherlands). Equal loading was confirmed using an anti-β-actin antibody (Sigma Chemical Co.). Quantitative Western blot analysis was performed using secondary goat anti-rabbit IRDye 680 and goat anti-mouse IRDye 800 CW with the Odyssey Scanner (LI-COR Biosciences, Lincoln, Nebraska USA) according to the manufacturer's instructions.

### Immunofluorescence

For immunofluorescence staining, cells were grown on coverslips overnight. Cells were fixed with ice-cold methanol for 30 minutes, washed twice with PBS, quenched with 20 mmol NH_4_Cl, and permeabilised with 0.1% Triton X-100 the following day. Cells were then incubated in blocking solution (PBS containing 3.0% BSA) for 45 minutes followed by incubation for 1 hour with anti-α-SMA antibody (Sigma Chemical Co.) diluted 1:100 in blocking solution. After washing, the labelled secondary antibody Alexa Fluor 488 goat anti-mouse immunoglobulin G (IgG) (Invitrogen) was used. Nuclei were stained using Hoechst 33258 (Invitrogen) according to the manufacturer's instructions. Specimens were visualised by using an Olympus IX51 inverted microscope at ×100 magnification using the cell^F ^Soft Imaging System (Olympus, Zoeterwoude, The Netherlands).

### Immunocytochemistry

Cells were cultured overnight on coverslips. The next day fixation in acetone followed by staining for α-SMA (α-SMA/1, M851, 1A4 clone; Dako, Carpinteria, CA, USA) at 1:500 dilution was performed for 60 minutes. Endogenous peroxidase was quenched with 0.1% natriumazide/0.3% hydrogen peroxide in PBS. After post-antibody blocking, goat poly-horseradish peroxidase (HRP) anti-mouse IgG (Immunologic, Duiven, The Netherlands) was added for 30 minutes. The colouring reaction was developed with 3-amino-9-ethylcarbazole (AEC), and counterstaining was performed with H & E.

### Immunohistochemistry

Paraffin-embedded tissue samples of 5-μm thickness were sequentially cut. Before blocking endogenous peroxidase activity with 1% hydrogen peroxide (Merck, Amsterdam, The Netherlands) in 2% PBS, sections were deparaffinised and rehydrated using xylene and a descending alcohol series. Blocking was performed with the following sequence: 2.5% periodic acid, 0.02% sodium borohydride and Protein Block (Dako).

Detection of TGF-β3: After deparaffinisation, antigen retrieval was performed in citrate buffer. Blocking was done with Protein Block (Dako) for 20 minutes. TGF-β3 antibody (Abcam) was applied overnight in a humid chamber at 4°C. Slides were rinsed in PBS, after which biotinylated link antibody was added (LSAB2 System; Dako) for 60 minutes. After slides were washed in PBS, streptavidin conjugate (LSAB2 System) was applied for 60 minutes.

Detection of P-Smad2: Prior to the application of Protein Block for 20 minutes, sections were pretreated with proteinase K (2 μg/mL in PBS) at 37°C for 30 minutes. P-Smad2 (Ser465/467; Cell Signaling Technology) was added overnight in a humid chamber at 4°C. Slides were rinsed in PBS, after which biotinylated link antibody was added (LSAB2 System) for 60 minutes. After PBS washing, streptavidin conjugate (LSAB2 System) was applied for 60 minutes.

Detection of α-SMA: After quenching endogenous peroxidase activity with 0.3% H_2_O_2 _in methanol, slides were heated in Tris-EDTA for 10 minutes at 100°C for antigen retrieval. The α-SMA antibody (α-SMA/1, M851, 1A4 clone; Dako, Glostrup, Denmark) was applied for 60 minutes followed by post-antibody blocking (Immunologic) for 15 minutes. After rinsing, goat poly-HRP against mouse IgG (Immunologic) was added for 30 minutes followed by PBS washing. All colouring reactions were developed by using 3,3'-diaminobenzidine (Sigma Chemical Co.) followed by counterstaining with H & E. Unlabelled samples were scored by an independent pathologist. Scoring was rated as follows: no staining (-) (except for staining in blood vessel walls), weak staining (+), moderate staining (++) and intense staining (+++).

Detection of P-ERK1/2: Before blocking endogenous peroxidase activity with 40% methanol and 1% H_2_O_2 _(Merck) in PBS, sections were deparaffinised and rehydrated using xylene and a descending alcohol series. Antigen retrieval using proteinase K (2.5 μL in 100 mmol Tris, pH 9.0, and 50 mmol EDTA, pH 8.0) for 10 minutes at 37°C was followed by three washes with 0.1 mol Tris-buffered saline (pH 7.4) containing 0.02% Tween 20 (TBST). Thereafter slides were incubated in 0.5% (Boehringer, Ingelheim, Germany) blocking reagent (BMP) in TBST for 60 minutes at 37°C. Subsequently, the P-ERK1/2 antibody (1:100; Cell Signaling Technology) diluted in 0.5% BMP/TBST was applied overnight at 4°C. Next, a species-specific biotinylated anti-IgG antibody (1:600 dilution in 0.5% BMP/TBST) was applied, followed by 45 minutes at 37°C. Incubation with streptavidin-HRP (1:200 dilution in 0.5% BMP/TBST) for 30 minutes at 37°C preceded and followed an amplification step using biotinyl-tyramide. Staining was carried out using AEC (Sigma Chemical Co., Zwijndrecht, The Netherlands) and Mayer's haematoxylin (Merck) according to the manufacturers' instructions. A water-based mounting solution was applied, and staining was visualised by using an Olympus IX51 inverted microscope equipped with the cell^F ^Soft Imaging System (Olympus). Unlabelled samples were scored by an independent researcher.

### Fibroblast-populated collagen lattice contraction assay

Three-dimensional fibroblast-populated collagen lattice (FPCL) contraction assays were carried out with primary cell cultures from passages 4 through 6. The assay was performed as described previously by others, with some modifications [30-32]. The collagen lattices were prepared by mixing a neutralising solution of COL1 (eight parts 3 mg/mL COL1 (PurCol^®^, Daiichi Sankyo, Parsippany, New Jersey, USA) one part 10 × α-MEM (Invitrogen) and one part HEPES (4-(2-hydroxyethyl)-1-piperazineethanesulfonic acid) buffer, pH 9.0). Final collagen and cell concentrations were adjusted to 2 mg/mL and 86 × 103 cells/mL using PBS, respectively. The cell-collagen mixture was aliquoted into PBS + 2% BSA-pretreated 24-well culture dishes (0.5 mL/well) and left to polymerise for 1 hour at 37°C. In each well, to the top of the polymerised lattice, we added 0.5 mL/well of DMEM containing 10% FBS. After 2 days of incubation at 37°C, the attached FPCLs were mechanically released from the sides of the culture plates, and fresh media supplemented with 0.5% FBS and the indicated substances were added. Images were obtained at various time points over a 5-day period using the Odyssey Scanner (LI-COR Biosciences). Collagen lattice areas were measured using the corresponding Odyssey 2.1 software.

### Statistical analysis

Values are expressed as means ± standard error of the mean. For statistical comparisons of two samples, an unpaired, a two-tailed Student's *t*-test with distinction of equal and unequal variances in a group (Levene's test) was used to determine the significance of differences between means. In addition, a nonparametric Mann-Whitney *U *test under the null hypothesis that the distributions of both groups (control versus Dupuytren-derived fibroblasts) were equal was performed for the data set shown in Figure 2C. All of the relevant comparisons were considered to be significantly different at *P *< 0.05. Experiments were performed at least three times, and representative results are shown.

## Results

### TGF-β/Smad signalling upregulated in DD

**Figure 1:**
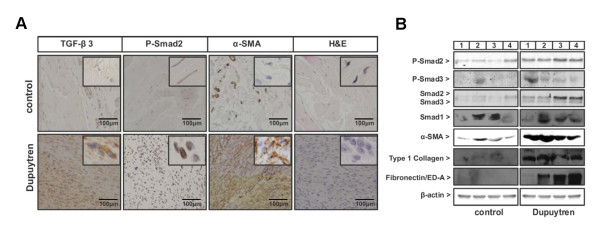
**Characterisation of Dupuytren's disease and control tissue specimens. (A)** Immunostaining of Dupuytren's disease (DD) and control tissue specimens for transforming growth factor β3 (TGF-β3), phosphorylated Smad2 (P-Smad2), α-smooth muscle actin (α-SMA) and H & E. Representative staining from DD patient 4 and control patient 1 is shown. Insets within each image are higher-magnification views. Evaluation of all four control and DD tissue samples are described in Table 2. **(B) **Protein expression analysis for P-Smad2; phosphorylated Smad3 (P-Smad3); total Smad2, Smad3 and Smad1; α-SMA; type I collagen; and fibronectin/ED-A by Western blot analysis of 100 μg of total protein extract isolated from four individual control patient-derived and four individual DD patient-derived tissue samples. β-actin was used as a loading control.

To evaluate the presence of TGF-β signalling in DD, nodules from the palmar fascia of four DD patients were surgically removed and compared to normal palmar fascia from four control patients who had undergone carpal tunnel release surgery (Table 1). Previous studies had shown an increase in TGF-β1 levels in DD; we extended these studies by examining TGF-β3, and also examined P-Smad2 as a measure for active canonical TGF-β signalling and α-SMA as a marker for myofibroblasts. Immunohistochemical staining of the normal fascia revealed weak TGF-β3 and P-Smad2 signals and no α-SMA expression (except for pericytes in blood vessels). This finding is in contrast to the tissues derived from DD patients, which displayed strong staining for TGF-β3, P-Smad2 and α-SMA. A high viable cell density, which is indicative of the proliferative stage of the cords, was confirmed with H & E staining (Figure 1A and Table 2).

**Table 1:** Overview of patients' details

Patients	Age, years	Gender	Tissue
Control 1	63	Female	Palmar fascia
Control 2	32	Female	Carpal ligament
Control 3	67	Male	Palmar fascia
Control 4	38	Female	Palmar fascia
Dupuytren 1	56	Female	Palmar fascia
Dupuytren 2	43	Male	Palmar fascia
Dupuytren 3	71	Male	Palmar fascia
Dupuytren 4	47	Male	Palmar fascia

**Table 2:** Evaluation of immunohistochemical staining^a^

Patients	TGF-β3	P-Smad2	α-SMA
Control 1	+	++	-
Control 2	++	++	-
Control 3	+	++	-
Control 4	+	-	-
Dupuytren 1	+++	++	++
Dupuytren 2	+++	+++	+++
Dupuytren 3	+++	+++	+++
Dupuytren 4	+++	++	+++

^a^Evaluation of immunohistochemical staining was performed on four control and four Dupuytren tissue samples. Positivity was graded on a scale from (-) to (+++), with (-) representing no staining, (+) representing weak staining, (++) representing moderate staining and (+++) representing strong staining. Staining in blood vessels was not included. The scoring was performed on unlabelled samples by a pathologist not involved in the study. Representative images are shown in Figure 1C.

Tissue samples were further investigated for active TGF-β signalling and for protein expression of key ECM components induced during fibrogenesis (Figure 1B). On average, Smad2 and Smad3 protein expression levels were significantly upregulated in DD patients compared to β-actin protein expression levels. Furthermore, we detected an increase in P-Smad2, but not P-Smad3, when normalised to total Smad2 and Smad3, respectively, in DD patients versus controls (Figure 1B and Additional file 1, Supplementary Figure 1). In contrast, Smad1 protein expression levels did not differ between control and DD patient material. P-Smad1 was not detected in control or DD samples (data not shown). Fibrogenesis ECM markers, such as COL1 and fibronectin/ED-A, were detectable in DD tissue but not in control samples. The myofibroblast marker α-SMA was strongly upregulated in all four DD patients (Figure 1B).

**Figure 2:**
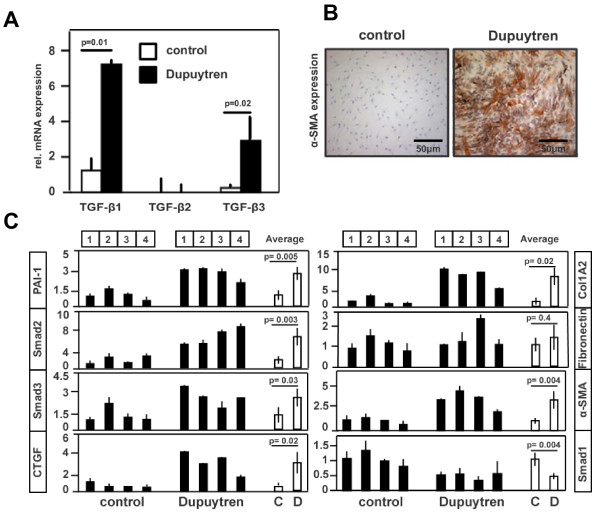
**Characterisation of primary Dupuytren’s fibroblasts and control fibroblasts. **(A)  Expression of TGF-β1, TGF-β2, and TGF-β3 mRNA in Dupuytren’s fibroblasts and control fibroblasts is  shown relative to glyceraldehyde 3-phosphate dehydrogenase (GAPDH) mRNA expression. Analysis  was done by quantitative PCR. RNA input for Dupuytren’s fibroblasts and control cDNA synthesis  consisted of an equal mixture of RNA derived from all four Dupuytren’s fibroblasts (mixture 1 through 4)  and all four control fibroblasts (mixture 1 through 4), respectively. Values are expressed relative to the  average of control (mixture 1 through 4) TGF-β1 mRNA values. (B) Immunostaining of Dupuytren’s and  control fibroblasts for α-SMA expression. Representative staining is shown for fibroblasts derived from  Dupuytren’s patient 4 and control patient 1. (C) Expression of plasminogen activator inhibitor 1 (PAI-1),  Smad2, Smad3 and connective tissue growth factor (CTGF) mRNA in Dupuytren’s and control  fibroblasts derived from four individuals each is shown relative to GAPDH mRNA expression. Analysis  was done by quantitative PCR. Individual control (1 through 4) and Dupuytren’s (1 through 4) mRNA  values are shown as well as their average values. Thus the average of mRNA expression of the  Dupuytren-derived fibroblasts is stated relative to the average of the control values.

We next examined whether primary fibroblasts derived from the tissue samples described above had similar properties. We first investigated the presence of all three TGF-β isoforms. In particular, the mRNA expression of the TGF-β1 and TGF-β3 isoforms was significantly upregulated in primary fibroblasts derived from DD tissue samples, whereas TGF-β2 mRNA expression was barely detectable (Figure 2A). Consistent with the results of the immunohistochemistry performed on the tissue samples, cultured Dupuytren's fibroblasts stained positive for α-SMA protein expression, whereas the control fibroblasts contained only very little α-SMA protein expression. The percentages of myofibroblasts in DD versus control patients was 40% to 50% versus 2% to 5% (Figure 2B).

We then quantitatively compared the mRNA expression levels of components involved in TGF-β signalling and fibrosis. On average, a nonparametric Mann-Whitney *U *test (data not shown) followed by an unpaired Student's *t*-test revealed that *Smad2 *and *Smad3 *mRNA expression, as well as expression of the TGF-β target genes *PAI-1 *and *CTGF*, were significantly upregulated in Dupuytren's fibroblasts compared to control fibroblasts. mRNA expression of the ECM component COL1, α2 (*COL1A2*) gene and the cytoskeleton representative α-SMA were also significantly increased, whereas the expression of fibronectin mRNA did not differ from that of control cells. The BMP signalling intracellular component Smad1 was present at lower levels in Dupuytren cells compared to normal fascia-derived cells (Figure 2C). The fact that the null hypothesis of the Mann-Whitney *U *test of equal distribution of control (patients 1 through 4) and Dupuytren-derived (patients 1 through 4) fibroblasts was rejected in 87.5% of the tested samples because we concluded that both control and Dupuytren-derived fibroblasts have an independent mRNA expression profile that also allows for statistical comparison, which furthermore allows the statistical analysis of pooled cell samples. Taken together, these results suggest that TGF-β/Smad signalling is increased in this fibroproliferative disease.

### SB-431542 inhibited fibrogenic properties of Dupuytren's fibroblasts

**Figure 3:**
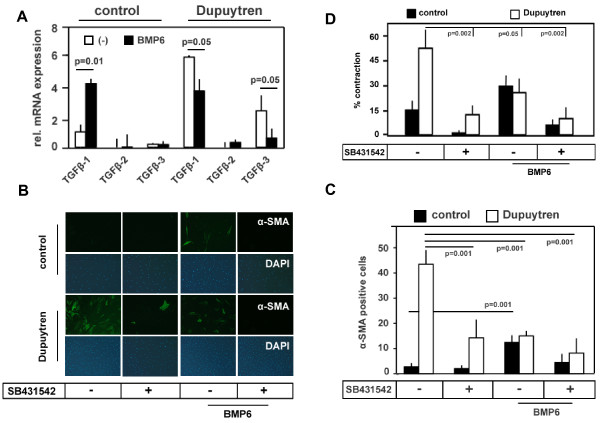
**Effects of SB-431542 and bone morphogenetic protein 6 on Dupuytren's and control fibroblasts**. **(A) **Quantitative PCR was used to determine the average expression of *TGF-β1, TGF-β2 *and *TGF-β3 *mRNA from control (mixture 1 through 4) and Dupuytren's (mixture 1 through 4) fibroblasts relative to *GAPDH *mRNA expression in the presence or absence of 100 ng/mL bone morphogenetic protein 6 (BMP6) for 18 hours. All values are expressed relative to the average of the control (1 through 4) *TGF-β1 *mRNA values. **(B) **Immunofluorescence of α-SMA in control and Dupuytren's fibroblasts treated for 72 hours with 20 μmol SB-431542 (+) in combination with or without 100 ng/mL rec. BMP6. Dimethyl sulfoxide (DMSO)-treated cells (-) were used as an internal control. Alexa Fluor 488 (green), α-SMA; 4',6-diamidino-2-phenylindole (blue), DNA. Representative staining is shown for Dupuytren's patient 4 and control patient 1. **(C) **Quantification of α-SMA-expressing control (mixture 1 through 4) and Dupuytren (mixture 1 through 4) cells after treatment with SB-431542 (20 μmol) in the presence or absence of rec. BMP6 (100 ng/mL) for 72 hours as depicted in Figure 3B. **(D) **Fibroblast-populated collagen lattice (FPCL) of control (1 through 4) and Dupuytren's (1 through 4) fibroblasts treated with DMSO (-) or 20 μmol SB-431542 (+) in the presence or absence of 100 ng/mL rec. BMP6. Quantification of the average contractions of control (1 through 4) and Dupuytren's (1 through 4) fibroblasts was calculated after 72 hours of treatment. Representative images are shown in Additional file 2, Supplementary Figure 2.

Because TGF-β signalling was proposed to play an important role in the etiopathogenesis of DD, we investigated the expression of TGF-β isoforms and the involvement of TGF-β-like signalling in the fibrogenic characteristics of the disease. We observed that TGF-β1 and TGF-β3 mRNA were expressed at much higher levels in Dupuytren's than in control fibroblasts (Figure 3A), and we noted a strong reduction in the elevated α-SMA expression in Dupuytren's fibroblasts upon treatment with SB-431542 (Figures 3B and 3C). Importantly, SB-431542 had strong inhibitory effects in the collagen contraction assay on both control and Dupuytren's cells (Figure 3C and Additional file 2, Supplementary Figure 2). Our data indicate that the self-induced basal contraction of Dupuytren's cells was caused by increased endogenous TGF-β-like Smad signalling, which enhanced α-SMA expression and promoted collagen contraction (Figure 3).

### BMP6 attenuated TGF-β signalling in Dupuytren's fibroblasts

Because it has been suggested that BMPs, particularly BMP7, can counteract TGF-β-induced fibrosis in the kidney, lung and liver, we investigated the effect of BMPs on Dupuytren's fibroblasts. BMP6, but not BMP7, attenuated endogenous TGF-β-like signalling. Quantitative PCR revealed that BMP6 strongly induced TGF-β1 mRNA expression in control cells but left the expression of the TGF-β2 and TGF-β3 isoforms unaffected (Figure 3A). In contrast to the control cells, in Dupuytren's fibroblasts BMP6 counteracted *TGF-β1 *and *TGF-β3 *mRNA expression (Figure 3A) and reduced *SMAD2 *and *SMAD3*, but not *SMAD1*, mRNA expression (data not shown).

As predicted on the basis of its antagonistic effects on TGF-β-like signalling, BMP6 (but not BMP7) attenuated α-SMA expression and counteracted the spontaneous elevated contraction seen in Dupuytren's fibroblasts (Figures 3B to 3D and Additional file 2, Supplementary Figure 2; data not shown). This inhibitory effect of BMP6 was further potentiated by simultaneous treatment with SB-431542 (Figures 3C and 3D and Additional file 2, Supplementary Figure 2).

### ERK1/2/MAP kinase signalling elevated in DD

**Figure 4:**
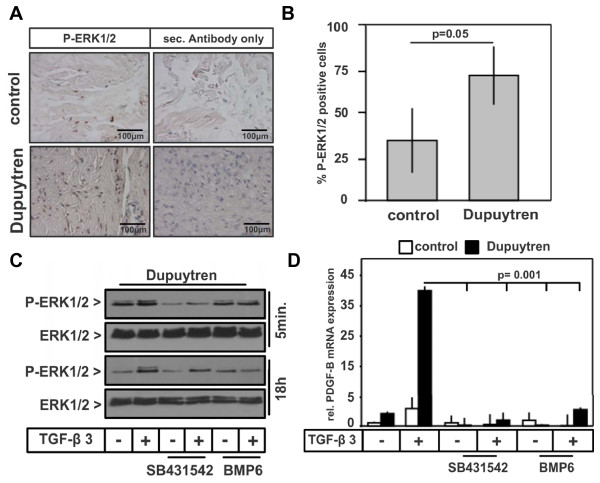
**Elevated phosphorylated extracellular signal-regulated kinase 1/2 expression in Dupuytren's tissue samples**. **(A) **Immunostaining for phosphorylated extracellular signal-regulated kinase 1/2 (P-ERK1/2) and secondary antibody control (P-ERK1/2 antibody omitted) of Dupuytren's and control tissue specimens. Representative staining from Dupuytren's patient 4 and control patient 1 is shown. **(B) **Quantitative analysis of P-ERK1/2-positive cells (shown in Figure 4A) from three control and three Dupuytren's tissue samples expressed as percentages. **(C) **Western blot analysis of lysates from pooled Dupuytren's fibroblasts (mixture 1 through 4) to determine P-ERK1/2 in untreated or rec. TGF-β3 (0.1 ng/mL)-treated cells for either 5 minutes or 18 hours in the presence or absence of SB-431542 (20 μmol) or rec. BMP6 (100 ng/mL). **(D) **Expression of *PDGF-B *(platelet-derived growth factor B) mRNA from four controls (mixture 1 through 4) and Dupuytren's fibroblasts (mixture 1 through 4) stimulated with 0.1 ng/mL rec. TGF-β3 in the presence or absence of 20 μmol SB-431542 or 100 ng/mL rec. BMP6 for 18 hours. Quantitative PCR was performed, and values are expressed relative to the average of the control (mixture 1 through 4) mRNA values using GAPDH as an internal reference.

**Figure 5:**
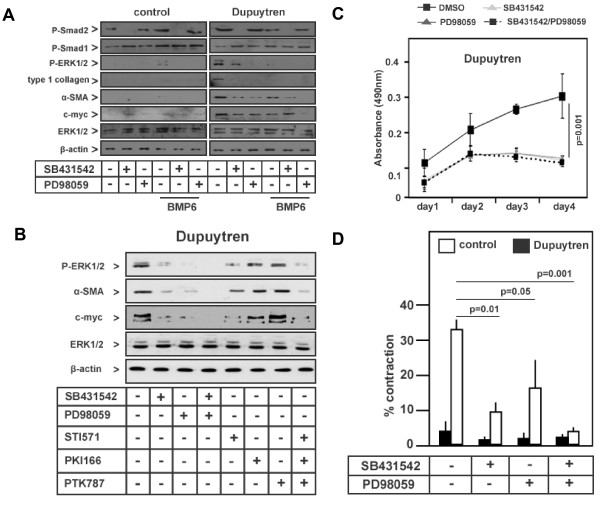
**TGF-β-induced ERK/mitogen-activated protein kinase signalling in Dupuytren's disease is inhibited by selective low-molecular-weight kinase inhibitors and BMP6**. **(A) **Western blot analysis for phosphorylated Smad2 (P-Smad2), P-Smad1, P-ERK1/2, type I collagen, α-SMA, c-myc and total ERK1/2 using specific antibodies on pooled control (mixture 1 through 4) and Dupuytren's (mixture 1 through 4) fibroblasts treated with SB-431542 (20 μmol), mitogen-activated protein kinase kinase 1 (MEK1) inhibitor PD98059 (10 μmol) and rec. BMP6 (100 ng/mL) for 18 hours. **(B) **Western blot analysis for P-ERK1/2, α-SMA, c-myc and total ERK1/2 using specific antibodies on pooled Dupuytren's fibroblasts (mixture 1 through 4) treated with SB-431542 (20 μmol), MEK1 inhibitor PD98059 (10 μmol), PDGF receptor (STI571, also called imatinib mesylate), epidermal growth factor receptor (PKI166) and vascular endothelial growth factor receptor (PTK787/ZK 222584) kinase inhibitors (10 μmol) for 18 hours. **(C) **3-(4,5-dimethylthiazol-2-yl)-5-(3-carboxymethoxyphenyl)-2-(4-sulfophenyl)-2H-tetrazolium (MTS)-based proliferation assay with pooled Dupuytren's fibroblasts (mixture 1 through 4) treated for 4 days with SB-431542 (20 μmol) and/or the MEK1 inhibitor PD98059 (10 μmol). Absorbance at 490 nm was measured daily, and the proliferation rate is stated relative to untreated Dupuytren's fibroblasts (mixture 1 through 4) at day 0. **(D) **FPCL on pooled control (mixture 1 through 4) and Dupuytren's (mixture 1 through 4) fibroblasts treated with SB-431542 (20 μmol) and/or PD98059 (10 μmol) for 72 hours.

It has been shown that TGF-β can activate non-Smad signalling pathways, such as MAP kinase signalling [9,10]. In addition, MAP kinases are activated by growth factors such as PDGF that have been implicated in DD [33,34]. We therefore investigated the phosphorylation of p38, c-Jun N-terminal kinase (JNK) and ERK in control and Dupuytren's tissue samples as well as in primary cells. While we did not detect phosphorylation of p38 and JNK (data not shown), phosphorylation of ERK1/2 was significantly increased in Dupuytren's tissue samples compared to control samples (Figures 4A and 4B). Similar results were obtained with fibroblasts isolated from Dupuytren's and control patients (Figures 4C and 5A).

We next determined the direct effects of TGF-β on the phosphorylation of ERK1/2 in Dupuytren's fibroblasts. We found that 5 minutes of TGF-β3 treatment induced a further increase in the phosphorylation of ERK1/2, which was inhibited by SB-431542 to a level lower than the basal level (Figure 4C). The presence of BMP6, however, had only marginal effects on the direct TGF-β3-induced phosphorylation of ERK1/2 (Figure 4C). In addition to its direct effect, TGF-β3 also induced an increase in ERK1/2 phosphorylation after 18 hours of stimulation. Interestingly, while SB-431542 showed only marginal effects on this sustained activation, BMP6 strongly attenuated this effect after 18 hours (Figure 4C).

The sustained effect of TGF-β3 on ERK1/2 was likely indirect and may have occurred via the TGF-β-mediated induction of growth factors. Indeed, *PDGF-B *and *PDGF-A *mRNA expression in particular were significantly upregulated in Dupuytren's fibroblasts and were strongly induced by TGF-β3 treatment (Figure 4D and data not shown). SB-431542 compound or BMP6 counteracted the TGF-β-induced increase in *PDGF-B *mRNA expression (Figure 4D).

### Targeting of TGF-β type I receptor and ERK1/2/MAP kinase pathways in Dupuytren's fibroblasts

We next set out to determine whether the elevated TGF-β/Smad and MAP kinase signalling pathways were causally linked to an increase in the expression of key fibrotic and proliferation proteins by interfering with these pathways using the ALK4, ALK5 and ALK7 inhibitor SB-431542 [8,30], the MEK1 inhibitor PD98059 [35] and BMP6. Treatment of Dupuytren's fibroblasts with SB-431542 completely inhibited elevated basal P-Smad2 levels and also attenuated P-ERK1/2 levels. This suggests that these increased basal activities are due to TGF-β or TGF-β-like ligands that are secreted by Dupuytren's fibroblasts themselves. PD98059 also strongly inhibited elevated basal P-ERK1/2 levels and had no significant effect on P-Smad2 levels (Figure 5A). Both treatments were associated with decreased expression of fibrotic marker proteins such as COL1 and α-SMA and reduced expression of the proliferation marker c-myc proto-oncogene (Figure 5A). Both SB-431542 and PD98059 treatment also inhibited *COL1A2, CTGF *and *PAI-1 *gene expression (Additional file 3, Supplementary Figure 3). The inhibitory effects of SB-431542 or PD98059 were potentiated by cotreatment with BMP6 (Figure 5A). Cotreatment with SB-431542/BMP6 and PD98059/BMP6 combinations decreased the levels of P-ERK1/2, COL1 and α-SMA to undetectable levels in Dupuytren's cells, which also was seen in untreated control cells. The c-myc level was significantly downregulated by PD98059/BMP6 and reached the low levels observed in control cells (Figure 5A).

We found that TGF-β3 strongly induced *PDGF *(Figure 4D), which, via its receptor, can activate ERK1/2/MAP kinase signalling. To determine the role of PDGF signalling in the augmented ERK1/2 phosphorylation observed in DD, we treated Dupuytren's fibroblasts with a selective PDGF receptor tyrosine kinase inhibitor (STI571, also known as imatinib mesylate) [36] and compared its effect with the effects of the inhibitors SB-431542 and PD98059. EGF receptor and VEGF receptor tyrosine kinase inhibitors [37,38] were used as specificity controls for the PDGF receptor kinase inhibitor. The PDGF receptor kinase inhibitor led to strong but incomplete decreases in ERK1/2 phosphorylation and c-myc expression (Figure 5B). Its effect was weaker than cotreatment of Dupuytren's fibroblasts with SB-431542 and PD98059. The EGF and VEGF receptor kinase inhibitors showed only minor effects. We could find no significant inhibition of the elevated α-SMA expression upon challenge of Dupuytren's fibroblasts with STI561 (Figure 5B), however, which is consistent with previous findings that link PDGF to proliferation and not to a myofibroblast transdifferentiation response [39].

The inhibitory effects of PD98059 suggest that the ERK1/2 MAP kinase pathway plays an important role in the increased fibrotic characteristics of Dupuytren's fibroblasts compared to control fibroblasts. When we stimulated Dupuytren's fibroblasts with TPA, which activates ERK1/2/MAP kinase pathways (as well as other pathways), we found elevated α-SMA expression and collagen contraction (Additional file 4, Supplementary Figure 4). Thus, ERK/MAP kinase signalling may be sufficient to weakly mediate the fibroproliferative properties observed in Dupuytren's fibroblasts.

Taken together, our results indicate that both the TGF-β/Smad and ERK1/2 MAP kinase signalling pathways contribute to the fibrogenic responses of Dupuytren's fibroblasts. We therefore determined whether we could normalise the fibroproliferative characteristics of Dupuytren's fibroblasts by targeting TGF-β-like signalling and ERK1/2/MAP kinase with SB-431542 and the MEK1 inhibitor PD98059, respectively. Concurrent treatment of Dupuytren's fibroblasts with SB-431542 and PD98059 abrogated ERK1/2 phosphorylation as well as α-SMA and c-myc expression (Figure 5B). Consistent with this observation, we found that treatment with SB-431542 and/or PD98059 strongly inhibited the elevated basal proliferation of Dupuytren's fibroblasts and had only minor effects on the proliferation rate of normal fibroblasts (Figure 5C and Additional file 5, Supplementary Figure 5). The high spontaneous contraction rate in Dupuytren's fibroblasts was completely blocked by cotreatment with SB431542 and PD98059 (Figure 5D).

## Discussion

DD is a chronic, fibroproliferative disorder that is most likely induced by overactive cytokines such as TGF-β, which is thought to play a prominent role by stimulating Dupuytren's fibroblasts to produce excessive levels of ECM proteins and by promoting their contractile phenotype [1]. In line with the results of previous studies, we found that biopsies and fibroblasts derived from primary cultures from affected areas in patients with DD had elevated expression levels of TGF-β, in particular the TGF-β1 and TGF-β3 isoforms, and that this correlated with increases in the expression levels of SMA, CTGF, fibronectin and collagen in Dupuytren's fibroblasts compared to controls [18,21].

TGF-β can signal via the Smad signalling pathways. We observed that patients with DD showed elevated expression of Smad2 and Smad3, but not Smad1. Of note, whereas P-Smad2 levels were found to be elevated, this was not clear for P-Smad3 levels. Smad2 and Smad3 may have distinct roles. In a recent article, investigators demonstrated that Smad3 is a negative regulator of α-SMA expression and the activation of the myogenic program in the epithelium [40]. When we challenged Dupuytren's fibroblasts with SB-431542, which inhibits TGF-β-like signalling pathways, the expression of key fibrotic markers such as PAI-1, CTGF, α-SMA and COL1 was decreased. Previous characterisation of the promoters of these target genes showed that they are regulated in a Smad-dependent manner [41,42]. Moreover, application of SB-431542 revealed that the high amount of spontaneous contraction of Dupuytren's fibroblasts, when embedded in a collagen lattice, was caused by overactive TGF-β-like signalling. TGF-β receptor kinase inhibitors have been shown to inhibit fibrotic responses in other cells *in vitro *and *in vivo *[29].

In recent years, a strong link has been established between TGF-β-induced fibrosis and BMP expression and signalling. Challenging the fibrogenic properties of Dupuytren's fibroblasts with BMP6 inhibited the gene expression of TGF-β1 and TGF-β3 and their respective downstream Smad2 and Smad3 effectors. Whereas previous studies attributed antifibrotic effects to BMP7, a close homolog of BMP6 [28], we were unable to demonstrate this for Dupuytren's fibroblasts. One could speculate whether BMP6 could compete with TGF-β for the recruitment of distinct receptors, thereby limiting TGF-β activity. Our data suggest a novel level of cross-talk, as previous studies have suggested that BMPs had an inhibitory effect on the TGF-β/Smad pathway through the formation of mixed Smad1/5-Smad2/3 complexes [43,44]. It is interesting that BMP6 in particular had an antagonising effect on TGF-β-driven DD, because it has been shown that myofibroblast progenitor cells derived from patients with diabetes are deficient in BMP6 expression [45], and there is some evidence of a relationship between diabetes and DD [46]. In another study, BMP6 and BMP7 were found to have differential effects on chemotaxis via a Smad4-independent, phosphoinositide 3-kinase-dependent pathway [47]. It would be worthwhile to explore whether similar mechanisms are of relevance in Dupuytren's fibroblasts. Although BMP6 may inhibit fibrotic responses, in discussing it as a potential therapeutic agent, one needs to take into account BMP6's action on normal fibroblasts and its strong osteoinductive properties [48].

**Figure 6:**
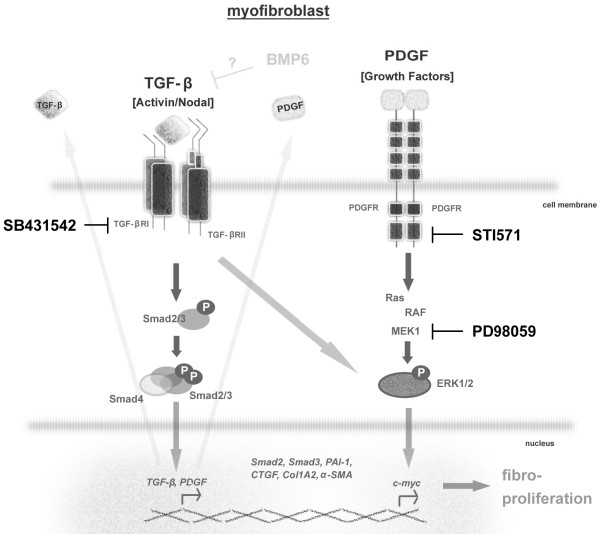
**Schematic representation of perturbed TGF-β/Smad and mitogen-activated protein kinase signalling pathways in Dupuytren's fibroblasts**. The TGF-β/Smad canonical Smad2/3 pathway and the noncanonical ERK1/2 mitogen-activated protein (MAP) kinase signalling pathways are depicted, as well as the PDGF-induced Ras/Raf/MEK/ERK1/2/MAP kinase pathway. TGF-β induces the production of PDGF and other growth factors. The symbols of corresponding downstream regulated genes are shown in italics. Inhibition by STI571, SB-431542 and PD98059 of the aberrantly overactivated signalling pathways in Dupuytren's fibroblasts is indicated. The mechanism by which BMP6 inhibits TGF-β expression and downstream responses is unclear. SB-431542 inhibits not only TGF-β/activin receptor-like kinase 5 (ALK5) signalling but also activin/ALK4 and nodal/ALK7 signalling. Our experiments do not rule out a role for these signalling pathways in Dupuytren's fibroblasts. Our data suggest that the TGF-β/Smad and PDGF/ERK MAP kinase pathways are targets for therapeutic intervention strategies to treat DD.

We found that Dupuytren's fibroblasts displayed overactive ERK1/2 signalling, but neither the JNK nor the p38 MAP kinase signalling pathway showed increased activity. This could be due to both direct TGF-β-induced ERK1/2 phosphorylation, since it was observed within 5 minutes and inhibited by SB431542, and indirectly through the induction of PDGF expression, which can stimulate ERK1/2 phosphorylation (schematically represented in Figure 6). Consistent with the latter idea, we found that treatment with the PDGF receptor inhibitor STI571 strongly mitigated the expression of phosphorylated ERK1/2.

The elevated ERK1/2 MAP kinase pathway could be linked to the elevated fibroproliferative characteristics of Dupuytren's fibroblasts. Treatment of cells with PD98059 inhibited the expression of fibrotic and proliferation markers. A role for MAP kinase signalling, also in cooperation with the Smad pathway, has been described for many TGF-β target genes [49-52]. In line with its potent inhibitory effects on fibroproliferative markers, spontaneous collagen contraction and elevated proliferation were inhibited by PD98059. Moreover, the finding that TPA induced ERK1/2 phosphorylation and collagen contraction suggests that activation of this pathway may be sufficient to induce contraction. BMP6 was not able to counteract this TPA-induced ERK response, which is in line with its proposed inhibitory actions further upstream at the level of TGF-β and Smad expression. Consistent with our results, inhibition of ERK1/2 MAP kinase signalling has been shown to mitigate fibrotic responses in scleroderma [53,54]. Our observations suggest a role for elevated PDGF signalling in promoting the proliferation of Dupuytren's fibroblasts. Of note, overactive PDGF signalling has been implicated in fibrosis in multiple tissues [55-57], and treatment with PDGF receptor kinase inhibitors has been shown to inhibit fibrosis [58,59].

Importantly, when both TGF-β receptors and ERK1/2 pathways were inhibited in Dupuytren's fibroblasts through simultaneous application of SB-431542 and PD98059, a complete block of the elevated basal proliferation and contraction was observed, which in turn commuted the Dupuytren's fibroblast phenotype into 'normal' fibroblasts.

## Conclusions

Both the TGF-β and ERK1/2 MAP kinase pathways cooperated in mediating the enhanced proliferation and high spontaneous contraction of Dupuytren's fibroblasts. Taken together, our data indicate that the TGF-β/Smad and ERK1/2 MAP kinase pathways are prime targets for the development of nonsurgical intervention strategies to treat patients with DD. For example, concurrent topical application of inhibitors such as SB-431542 and PD98059 into the DD area could block fibroproliferative responses and recurrence in DD while preventing the potential problems associated with systemic administration of such compounds (schematically represented in Figure 6).

## Abbreviations

BSA: Bovine Serum Albumin; DMEM: Dulbecco's Modified Eagle's Medium; H & E: Haematoxylin and Eosin Stain; PBS: Phosphate-Buffered Saline; PCR: Polymerase Chain Reaction.

## Competing interests

The authors declare that they have no competing interests.

## Authors' contributions

CK and PtD designed the experiments, analysed the data and wrote the manuscript. CK performed the experiments. PtD supervised the research. PK obtained the patient material and isolated primary fibroblasts. PK analysed the immunohistochemical experiments. All of the authors discussed the results and commented on the manuscript.

## Supplementary Material

Additional file 1 **Quantification of total Smad and phosphorylated Smad (P-Smad) protein expression levels as depicted in Figure 1B**. Quantification was performed by densitometric analysis using the Odyssey system (LI-COR Biosciences). All values are expressed relative to β-actin protein expression levels (C, Control; D, Dupuytren). Click here for file

Additional file 2 **Fibroblast-populated collagen lattice (FPCL) of controls' (1 through 4) and Dupuytren's patients' (1 through 4) fibroblasts treated with dimethyl sulfoxide (DMSO) (-) or 20 μmol SB-431542 (+) in the presence or absence of 100 ng/mL rec**. bone morphogenetic protein 6 (BMP6). Corresponding images demonstrating the quantification of contraction in Figure 3D are shown. Click here for file

Additional file 3 **Quantitative PCR was used to determine the average expression of *α-SMA, Smad1, Smad2, Smad3, PAI-1 *(plasminogen activator inhibitor 1), *fibronectin, CTGF, c-myc *and *COL1A2 *mRNA from control (mixture 1 through 4) and Dupuytren's (mixture 1 through 4) fibroblasts relative to *GAPDH *mRNA expression in the presence or absence of SB-431542 (20 μmol) and/or the MEK1 inhibitor PD98059 (10 μmol) and/or 100 ng/mL BMP6 for 18 hours**. All values are expressed relative to the average of the untreated control (1 through 4) mRNA values. Click here for file

Additional file 4 **Top: FPCL on control (mixture 1 through 4) and Dupuytren's fibroblasts (mixture 1 through 4) treated with 12-*O*-tetradecanoylphorbol-13-acetate (TPA) (100 nmol) in the presence or absence of rec**. BMP6 (100 ng/mL) for 72 hours. Middle: Representative images of each condition are shown. Bottom: Western blot analysis of phosphorylated extracellular signal-regulated kinase 1/2 (P-ERK1/2) and α-smooth muscle actin (α-SMA) on primary control (mixture 1 through 4) and Dupuytren's fibroblasts (mixture 1 through 4) treated with TPA (100 nmol) in the absence or presence of rec. BMP6 (100 ng/mL) for 18 hours are depicted in the lower panel. β-actin was included as a loading control. Click here for file

Additional file 5 **3-(4,5-dimethylthiazol-2-yl)-5-(3-carboxymethoxyphenyl)-2-(4-sulfophenyl)-2H-tetrazolium (MTS)-based proliferation assay of pooled control fibroblasts treated for four days with SB-431542 (20 μmol) and/or the mitogen-activated protein kinase kinase 1 (MEK1) inhibitor PD98059 (10 μmol) where indicated**. Absorbance at 490 nm was measured daily, and the proliferation rate is stated relative to untreated cells at day 0. Click here for file
