# Supplementary figures and images for: Elevated transforming growth factor β and mitogen-activated protein kinase pathways mediate fibrotic traits of Dupuytren's disease fibroblasts
Source: Fibrogenesis Tissue Repair. 2011 Jun 28;4:14. doi: 10.1186/1755-1536-4-14 (PMC3148569; doi:10.1186/1755-1536-4-14)

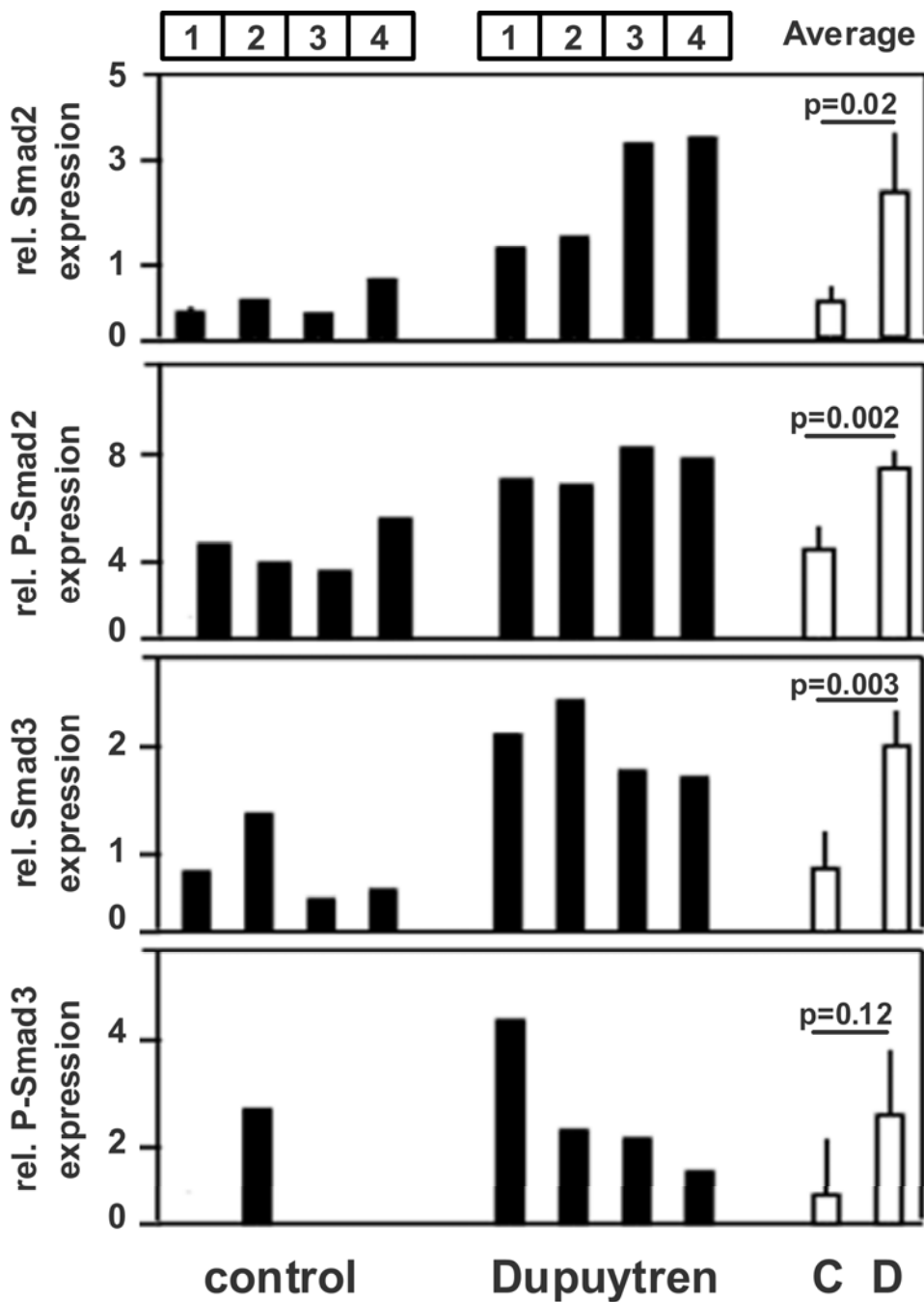

Supplement: Additional file 1 — Quantification of total Smad and phosphorylated Smad (P-Smad) protein expression levels as depicted in Figure 1B. Quantification was performed by densitometric analysis using the Odyssey system (LI-COR Biosciences). All values are expressed relative to β-actin protein expression levels (C, Control; D, Dupuytren). [file 1755-1536-4-14-S1.PDF]

|   |   |   |   |
|---|---|---|---|
| 1 | 2 | 3 | 4 |
|---|---|---|---|

|   |   |   |   |
|---|---|---|---|
| 1 | 2 | 3 | 4 |
|---|---|---|---|

DMSO >

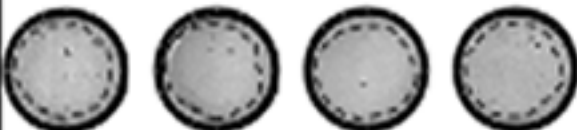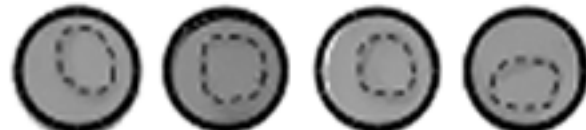

SB431542 >

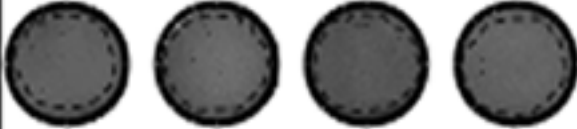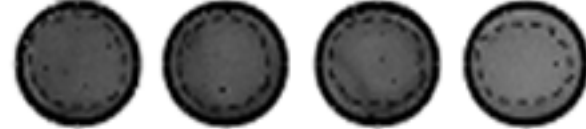

BMP6 >

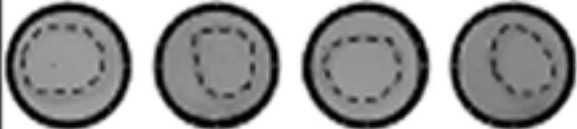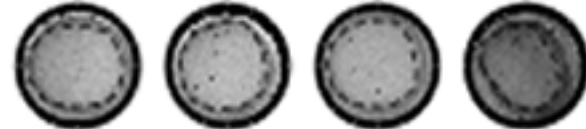

SB431542 >

+  
BMP6

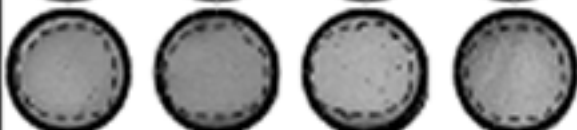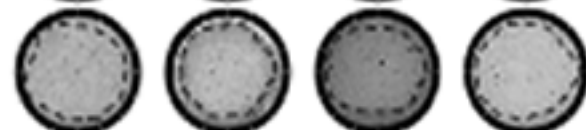

Supplement: Additional file 2 — Fibroblast-populated collagen lattice (FPCL) of controls' (1 through 4) and Dupuytren's patients' (1 through 4) fibroblasts treated with dimethyl sulfoxide (DMSO) (-) or 20 μmol SB-431542 (+) in the presence or absence of 100 ng/mL rec. bone morphogenetic protein 6 (BMP6). Corresponding images demonstrating the quantification of contraction in Figure 3D are shown. [file 1755-1536-4-14-S2.PDF]

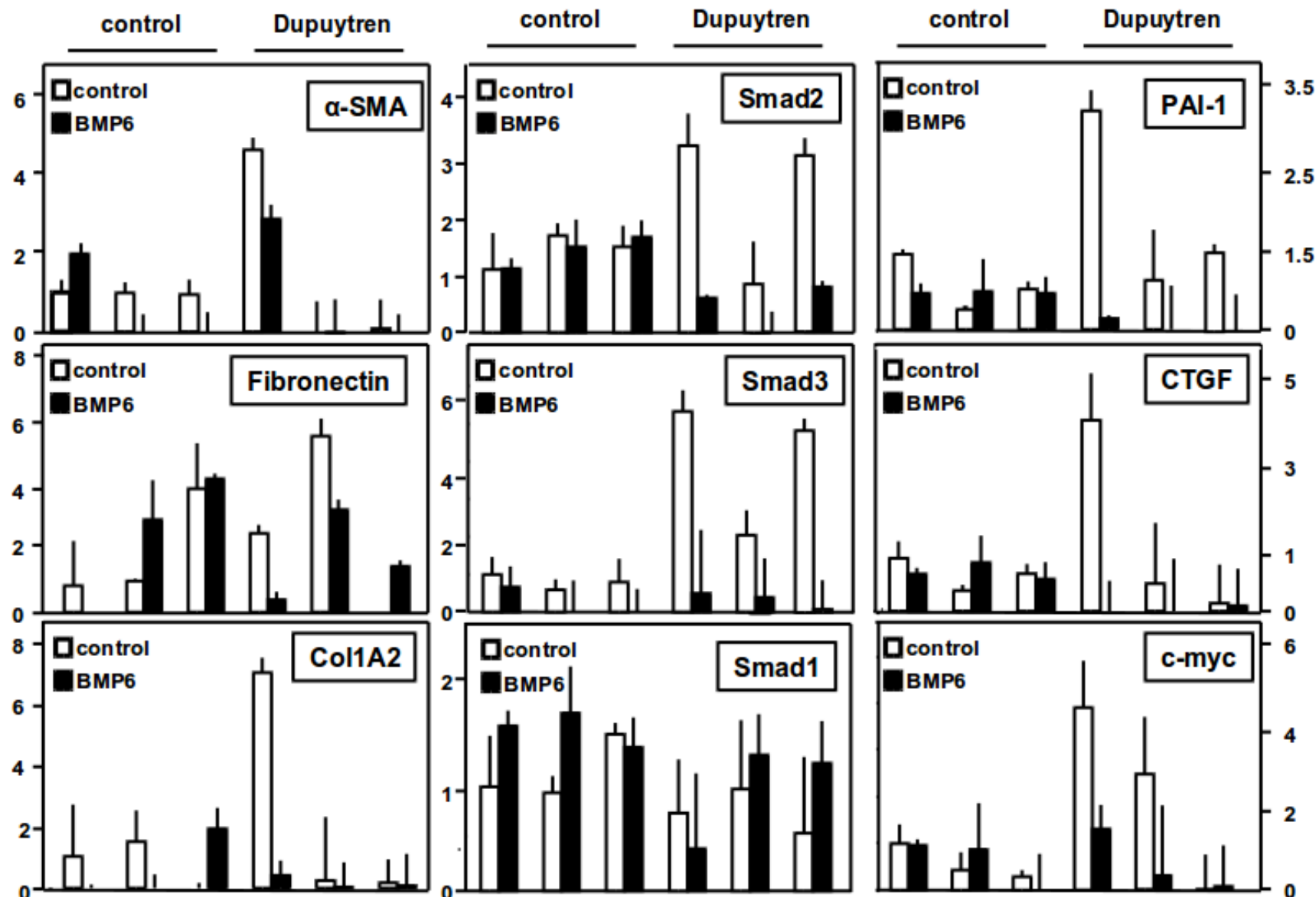

SB431542

-

+

-

-

+

-

-

+

-

+

-

PD98059

-

-

+

-

-

+

-

-

-

+

+

Supplement: Additional file 3 — Quantitative PCR was used to determine the average expression of α-SMA, Smad1, Smad2, Smad3, PAI-1 (plasminogen activator inhibitor 1), fibronectin, CTGF, c-myc and COL1A2 mRNA from control (mixture 1 through 4) and Dupuytren's (mixture 1 through 4) fibroblasts relative to GAPDH mRNA expression in the presence or absence of SB-431542 (20 μmol) and/or the MEK1 inhibitor PD98059 (10 μmol) and/or 100 ng/mL BMP6 for 18 hours. All values are expressed relative to the average of the untreated control (1 through 4) mRNA values. [file 1755-1536-4-14-S3.PDF]

**control**

**Dupuytren**

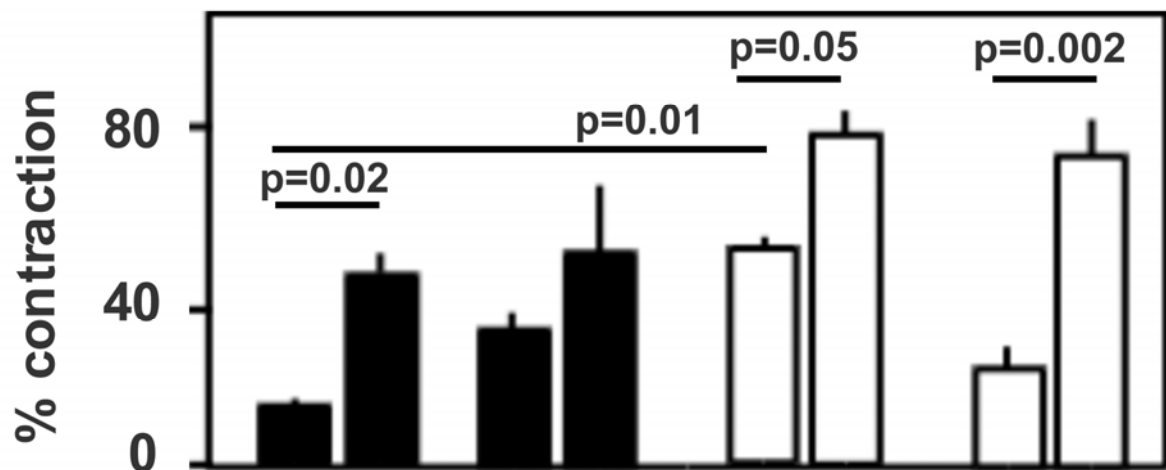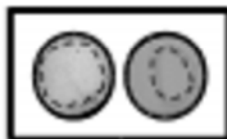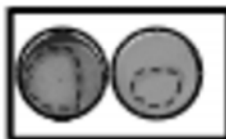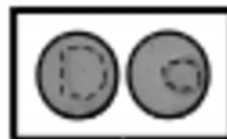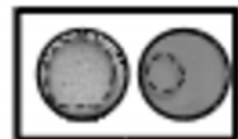

P-ERK1/2 >

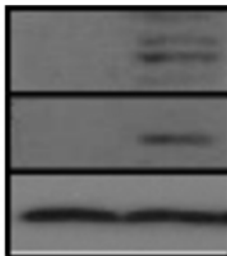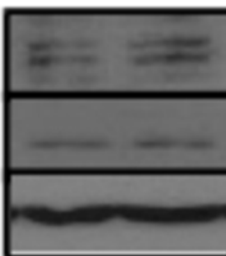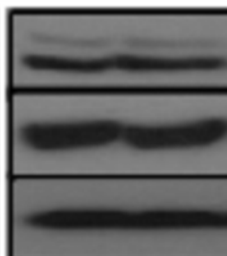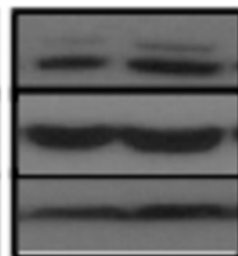

$\alpha$ -SMA >

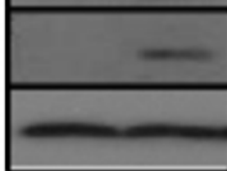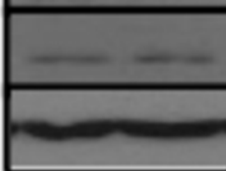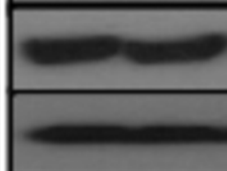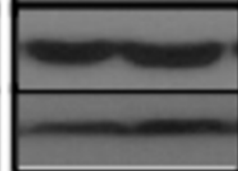

$\beta$ -actin >

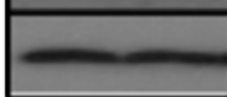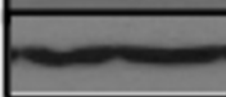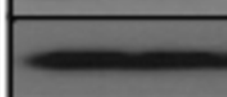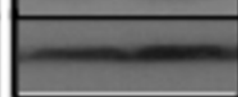

**TPA**

-

+

-

+

-

+

-

+

**BMP6**

**BMP6**

Supplement: Additional file 4 — Top: FPCL on control (mixture 1 through 4) and Dupuytren's fibroblasts (mixture 1 through 4) treated with 12-O-tetradecanoylphorbol-13-acetate (TPA) (100 nmol) in the presence or absence of rec. BMP6 (100 ng/mL) for 72 hours. Middle: Representative images of each condition are shown. Bottom: Western blot analysis of phosphorylated extracellular signal-regulated kinase 1/2 (P-ERK1/2) and α-smooth muscle actin (α-SMA) on primary control (mixture 1 through 4) and Dupuytren's fibroblasts (mixture 1 through 4) treated with TPA (100 nmol) in the absence or presence of rec. BMP6 (100 ng/mL) for 18 hours are depicted in the lower panel. β-actin was included as a loading control. [file 1755-1536-4-14-S4.PDF]

# control

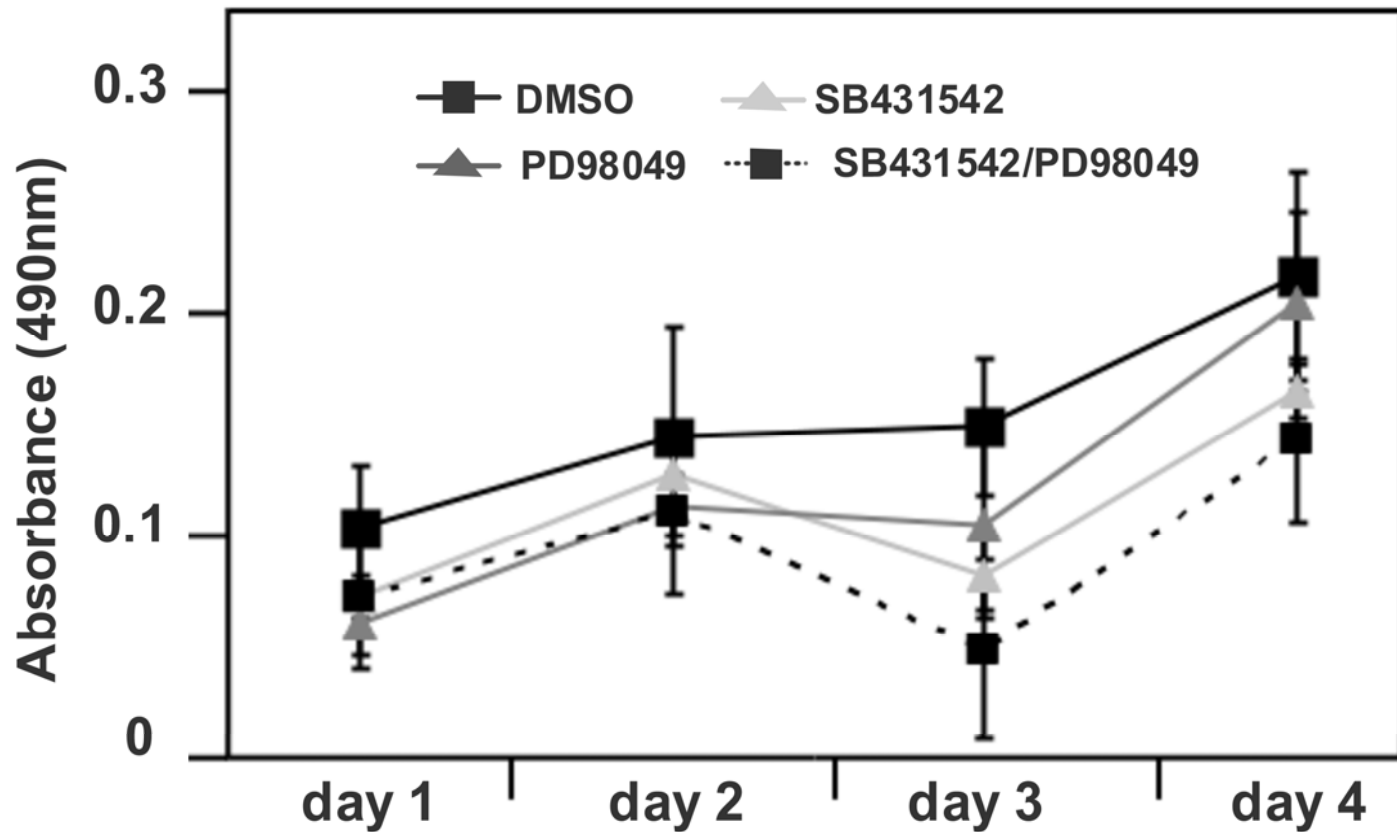

Supplement: Additional file 5 — 3-(4,5-dimethylthiazol-2-yl)-5-(3-carboxymethoxyphenyl)-2-(4-sulfophenyl)-2H-tetrazolium (MTS)-based proliferation assay of pooled control fibroblasts treated for four days with SB-431542 (20 μmol) and/or the mitogen-activated protein kinase kinase 1 (MEK1) inhibitor PD98059 (10 μmol) where indicated. Absorbance at 490 nm was measured daily, and the proliferation rate is stated relative to untreated cells at day 0. [file 1755-1536-4-14-S5.PDF]
